# Predictors of activity involvement in dementia care homes: a cross-sectional study

**DOI:** 10.1186/s12877-017-0564-7

**Published:** 2017-08-04

**Authors:** Dieneke Smit, Jacomine de Lange, Bernadette Willemse, Anne Margriet Pot

**Affiliations:** 1 Department of Clinical, Neuro and Developmental Psychology, Faculty of Behavioral and Movement Sciences, Vrije Universiteit Amsterdam, Van der Boechorststraat 1, 1081 BT Amsterdam, The Netherlands; 2 Program on Aging, Netherlands Institute of Mental Health and Addiction, PO box 725, 3500 AS Utrecht, the Netherlands; 3 Research Centre Innovations in Care, Rotterdam University of Applied Sciences, Rochussenstraat 198, 3015 EK Rotterdam, the Netherlands; 4 School of Psychology, University of Queensland, Brisbane, Australia

**Keywords:** Cross-sectional study, Dementia care homes, Predictors, Activity involvement

## Abstract

**Background:**

Despite the finding that involvement in activities is one of the most important needs of residents with dementia living in care homes, care facilities struggle to fulfill this need. Over the years, various factors are suggested which may contribute to or disable activity provision in dementia care homes. These include limited financial resources, task oriented staff and disease-related characteristics of residents. This study aims to further clarify which of these factors predict higher activity involvement.

**Methods:**

Data were derived from the second measurement (2011) of the Living Arrangements for people with Dementia study. One thousand two hundred eighteen people residing in 139 dementia care homes were involved. Forty predictors of higher involvement were studied. Multilevel backward regression analyses were performed.

**Results:**

The most important predictors of higher involvement were: absence of agitation, less ADL dependency, and a higher cognitive status of the residents, higher staff educational level, lower experienced job demands by care staff and a smaller number of residents living in the dementia care wards of a facility. More social supervisor support as perceived by staff was found to predict less activity involvement.

**Conclusions:**

To increase the activity involvement of care home residents with dementia it seems vital to: 1) reduce staff’s experienced job demands; 2) elevate their overall educational level; 3) train staff to provide suitable activities, taking account of the behavior and preserved capabilities of residents; and 4) foster transition towards small-scale care. In order to achieve these aims, care organizations might need to evaluate the use of their financial means.

**Electronic supplementary material:**

The online version of this article (doi:10.1186/s12877-017-0564-7) contains supplementary material, which is available to authorized users.

## Background

The involvement in activities by people with dementia living in long-term care homes is frequently associated with higher quality of life outcomes [1]. Several intervention studies have shown that involvement in recreational, vocational or leisure activities could increase positive mood or decrease behavioral symptoms during and directly after involvement, and might also have beneficial effects on these outcomes over time [2–4].

Moreover, activity provision is increasingly cited as an indicator of resident and family satisfaction with care [5]. A literature review reveals that besides the management of behavioral symptoms, involvement in meaningful activities and social interaction were the most important needs for long-term care residents with dementia - as described by care staff, family caregivers and persons with dementia themselves [6]. In a modern society, enabling people to do what they find of value is perceived as a basic human right for the aging population, including people with dementia [7].

Yet, despite these urgent calls for making activity involvement one of the core elements of long-term care provision, many dementia care homes struggle to reach an appropriate level of activity involvement among their residents [8–10]. The dementia care home is often described as a place of boredom where residents do little besides sleeping, eating, looking around, and having a conversation [11–13]. In our previous research, we found that on average, residents were involved in activities for less than 1 hour a day besides having a conversation, listening to music or the radio, or watching TV [14].

Knowing the barriers and facilitators of activity involvement in dementia care homes might help to find solutions for the unfulfilled need for activities among residents with dementia. Over the last two decades, many factors that predict activity provision in long-term dementia care have been suggested and studied (see Additional file 1 for an overview of the literature on predictors of activity involvement). These potential predictors of activity involvement can be grouped into characteristics of 1) *residents with dementia*, 2) *finances, staff ratio and staff educational level*, 3) *modern* versus *traditional care culture within the care home*, 4) *job strain as perceived by care staff*, 5) *the physical care environment*, and 6) *the organization of activities*. These are discussed briefly below.

Characteristics of the *residents with dementia* include disease-related characteristics and sociodemographic characteristics. Examples are physical and cognitive impairment, challenging behaviors (e.g. agitation, apathy, anxiety and depression) [15–25], age, gender and length of stay in the care home [16, 22, 26, 27].

With regard to the characteristics of *finances, staff ratio and staff educational level,* it is assumed that the limited financial resources available to care homes, resulting in a low staff ratio and a low staff educational level, or little knowledge of dementia, negatively impact the activity involvement of residents [12, 27–34]. A stable care team with sufficient knowledge of dementia care is likely to result in higher activity involvement [35], as well as the availability of professional treatment for residents (for example, assessment for depression by a mental health professional) [16].

Examples of characteristics of *a modern as opposed to a traditional care culture* in a care home, are the presence of a well implemented philosophy on quality care and a transformational leadership style. These factors were found to enhance activity provision [4, 36]. Delivering person-centered care - requiring staff to gain knowledge of the biography and psychological needs of the residents in order to adjust their approach and care to the individual care recipient – and family involvement [16, 17] are also mentioned as factors that stimulate activity provision in long-term dementia care [33, 37]. This contrasts with the traditional focus on routines, in which priority is given to care tasks over psychosocial needs [21, 27, 33, 38]. The traditional higher administration of psychotropic drugs and use of physical restraints for the treatment of challenging behavior are thought to negatively influence the activity involvement of residents [15, 16, 25].

*Job strain as perceived by care staff* [12, 16, 33, 35, 37] is the result of a complex combination of factors, such as the physical and emotional care needs of residents, staffing levels, support from colleagues and supervisor, decision authority, and the feeling of being competent to care for their care recipients [39, 40]. Examples of perceptions of strain that were found to result in limited activity provision to residents, are a lack of conviction of being capable of involving residents in activities [15, 17],and a perceived lack of support from supervisors and colleagues with regard to spending time on providing activities [28].

With regard to characteristics of the *physical care environment,* a small-scale group living home environment, or a recognizable or homelike environment with opportunities for residents to be engaged in normal household activities, was found to stimulate activity involvement in several studies [41–45].

*The organization of activities* refers to differences in the activities offered by care homes. Providing smaller and individual activities that are tailored to the needs, skills and preferences of residents seems to enhance their engagement in activities [24, 46–49]. Presumably, this should not solely be the task of activity or recreational staff [3, 16, 27, 28, 50]. Conversely, offering activities in the form of standard, centrally provided activity schedules for large resident groups is thought to predict lower activity involvement [15, 20, 51].

In conclusion, many factors may have a disabling or enabling impact on the involvement of people with dementia living in care homes. However, the factors mentioned in the literature are often only suggested, have been studied but not scientifically tested, or have been studied within small sample sizes. It is also unclear how the various factors relate to each other. In the current study, we further clarify how the following characteristics relate to higher activity involvement: characteristics concerning 1) *residents with dementia,* 2) *resources in terms of finances, staff ratio and educational level,* 3) *modern* versus *traditional care culture,* 4) *the job strain perceived by care staff*, 5) *the physical care environment* and 6) *the organization of activities*. The findings of this study may provide care organizations with pointers to address their residents’ need for occupation.

## Methods

### Design and sample

#### Design

**Fig. 1:**
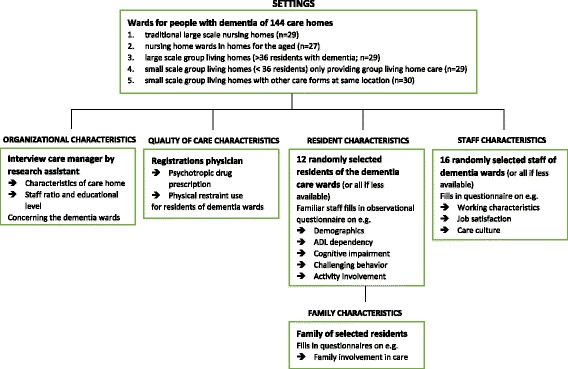
Design of the 2nd measurement cycle of the LAD-study

This study has a cross-sectional design. Observational data were used from questionnaires and interviews that were obtained in the second measurement cycle (January – June 2011) of the Living Arrangements for People with Dementia (LAD-) study. The LAD-study is an ongoing study on developments in Dutch nursing home care for people with dementia and the consequences of environmental and organizational characteristics - such as group living home care, person-centeredness and staffing levels - for residents, family and staff wellbeing. Data collection takes place every 2 years. The design of the first measurement cycle of this study has been described in detail elsewhere [52]. In Fig. 1, the design of the 2nd measurement cycle of the LAD-study is shown schematically.

The reason we used data from the second measurement cycle is that in the first cycle, solely data on residents’ involvement in types of activities were collected and not on time spent. Previous research pointed to the need to also collect data on time spent on these activities [44]. For this purpose, the measurement instrument was expanded in the second cycle.

#### Care home settings

Data from 144 long-term-care facilities providing nursing home care for people with moderate to very severe dementia were gathered. In the Netherlands, people with a primary diagnosis of dementia are cared for on dementia-specific care units or in dementia-specific care homes. The participating living arrangements represented the five types of nursing home care that are provided in the Netherlands: traditional large-scale nursing homes (*n* = 28), nursing home units in homes for the aged (*n* = 30), large-scale group-living homes (defined as group-living home care facilities with 36 or more residents with dementia; *n* = 28), ‘archetypal’ small-scale group-living homes (defined as fewer than 36 residents with dementia) that solely provided group-living home care (*n* = 28), and small-scale group-living homes that also provided other types of care at the same location, for instance care for somatic patients (*n* = 25). In the Netherlands, small-scale group-living homes for people with dementia are designed to provide person-centered long-term care, where residents can reside until death, despite severe cognitive or physical impairments. Previous research has shown however, that residents of ‘archetypal’ small-scale group-living homes were less physically and cognitively impaired than residents of large-scale nursing homes on average. Furthermore, small-scale group-living home residents were sometimes transferred to regular nursing homes when their care needs increased [53, 54]. The participating care homes were all state-financed.

#### Data collection procedure

In each participating care home, a care manager was interviewed by a trained research assistant to obtain data on environmental and organizational characteristics as well as staff ratio of the care homes’ care units for persons with dementia. In each care home, the old age care physician was asked to fill out registration forms on the prescription of physical restraints as well as psychotropic drugs from the medical records of all residents residing on the dementia care units.

To gather information on the activity involvement of residents with dementia, their physical and cognitive impairment, behavioral symptoms and demographics, 12 residents from the dementia care units were randomly selected in each care home. All residents on the participating dementia units were eligible to participate. If there were fewer than 12 residents of dementia care units in a home, all residents of these units were included. A registered nurse (RN) or certified nursing assistant (CNA) who was most involved with a selected resident was asked to complete observational questionnaires. For feasibility reasons, staff could not be trained in completing these questionnaires. Therefore, the questionnaires were provided with detailed instructions on how to answer the questions of the instruments used. Staff were also invited to contact the research group for assistance at any time.

To collect data on family satisfaction and involvement in their relative’s care, the primary family caregiver of each randomly selected resident was invited to participate in the LAD-study by completing a standardized questionnaire (see Additional file 2 for an overview of the measures used in this questionnaire).

Furthermore, 16 care staff members (i.e. nursing assistants, CNAs, RNs) who worked on the dementia care units were randomly selected in each care home to obtain information on working characteristics, job satisfaction and care culture. If there were fewer than 16 care staff members, all care staff were included. Only care staff working on a permanent basis were eligible to participate.

### Measures

#### Dependent variable: Involvement in activities

The activity involvement of residents with dementia was measured using the Activity Pursuit Patterns from the Resident Assessment Instrument Minimum Data Set (RAI-MDS [55]). To our knowledge, no explicit data on intra- and interrater reliability are available on the Activity Pursuit Patterns [56, 57]. The instrument consists of a list of 20 activities (Table 1) for which an RN or CNA retrospectively answers the question whether or not the resident has been involved in these activities during the past 3 days. To study the time of activity involvement, we expanded the original Activity Pursuit Patterns questionnaire by adding questions on how many times the person was involved in this activity during the past . days, and for how many minutes on average for each time.

**Table 1:** The 20 activities listed by the Activity Pursuit Patterns of the MDS-RAI and estimated time of involvement of study population (*n* = 1218) during three days

Activities MDS-RAI		Range time involved	Mean	SD
1	Playing cards, games, puzzles	0–420	18.25	44.60
2	Using the computer	0–90	0.16	3.35
*3*	*Talking or making a phone call* ^*a*^	*0–600*	*45.15*	*66.21*
4	Handwork or art	0–360	6.66	28.42
5	Dancing	0–120	1.61	9.70
6	Exercise or sports	0–180	9.82	21.04
7	Gardening, taking care of plants	0–120	1.07	7.59
8	Helping others	0–90	1.99	8.69
*9*	*Music or singing* ^*a*^	*0–540*	*30.91*	*52.92*
10	Pets	0–360	4.40	20.43
11	Reading, writing, cross-word puzzles	0–630	17.72	52.99
12	Spiritual or religious activities	0–360	14.76	35.82
13	Excursion or shopping	0–720	15.87	52.42
14	Take a walk outside	0–540	25.38	50.08
*15*	*Watching TV or listening to the radio* ^*a*^	*0–2100*	*140.43*	*205.93*
16	Domestic tasks	0–370	6.84	24.12
17	Cooking	0–300	5.57	19.31
18	Conversation groups	0–360	6.42	22.36
19	‘Snoezelen’ or sensory stimulation	0–420	5.34	22.57
20	Beauty activities (manicure, hairdressing, make-up)	0–240	9.74	19.30

^a^ excluded from analyses for reliability reasons (suspected confusion between passive and active involvement)

Estimated times that residents were involved in any of the listed activities during the past 3 days were calculated (Table 1). It was found that RNs and CNAs sometimes reported that residents were involved in talking, music or singing, or watching television (activity number 3, 9 and 15) for very long periods, sometimes the entire time they were awake. Further investigation taught us that in some cases a resident spoke to himself the whole day, or that residents sat in a place where the radio or television was on for several hours, without actual involvement in conversation, singing, watching a television program or listening to music. Since the purpose of this study was to analyze predictors of actual involvement in activities, these occupations were excluded from the analyses. The total duration of involvement was therefore calculated on the basis of the time residents were involved in the 17 remaining activities during the past 3 days.

#### Predictor variables

The predictor variables used in this study consisted of all variables that were measured in the LAD-study that represented factors that have been cited in the literature as influencing activity involvement of residents with dementia. Accordingly, 40 predictor variables were studied. In Table 2, these variables and the instruments that were used to obtain them are specified. Below, these are described further.

*1) Characteristics of residents with dementia*

**Table 2:** List of factors that were suggested to influence activity involvement of care home residents with dementia that were represented in the LAD-study; description of variables and measurement specifications

Proposed factor in literature	Selected variables in LAD-study	Measurement specifications
Characteristics of residents with dementia		
Age	1. Age	Years acquired by date of birth (42 to 101)
Gender	2. Gender	Male or female
Marital status	3. Having a life partner	Life partner yes or no/diseased
Length of stay	5. Length of stay	< 6 months or >6 months
ADL dependency	6. ADL dependency	Katz ADL dependency scale (1 to 7)
Immobility	7. Immobility	Transfer-item of Katz scale was dichotomized in mobile (being able to transfer independently with or without instrumental means) and immobile (hardly or not able to transfer independently)
Cognitive functioning	8. Cognitive impairment	Cognitive Performance Scale (CPS; 0 to 6)
Behavioral symptoms	9. Challenging behavior	Short version Neuropsychiatric Inventory questionnaire (NPI-Q; 0 to 36)
depression	10. Depression	The separate NPI-Q items for depression, agitation, anxiety, and apathy were dichotomized into no or rare occurrence, and frequent occurrence.
agitation	11. Agitation	
anxiety	12. Anxiety	
passivity, apathy (loss of interest or lack of motivation)	13. Apathy	
Characteristics of resources of finances, staff ratio and educational level		
Staff ratio / shortness of time and resources	14. Staffing levels	Hours of care staff available per week per resident on dementia care wards (13.86 to 30.48)
Insufficient knowledge of dementia / lack of skills / Formal staff training	15. Educational level of care staff	Percentage of scheduled care staff with education level 3 (certified nursing assistant) or higher (22.70 to 100)
Ratio receiving professional or unprofessional treatment / Involvement of physicians or paramedics in care planning	16. Availability of (para)medics	Number of available hours of (para)medic staff per week per resident of dementia care wards (0 to 5.06)
Instability of care team / High staff turnover / leave of skilled staff that were not replaced	17. Number of vacancies	Number of care staff vacancies per resident of dementia ward that were not yet fulfilled (0 to 0.22)
	18. Average sick leave	Average percentage of sick leave of the past 6 months concerning staff working on dementia care wards (0.82 to 17.0)
Characteristics of modern or traditional care culture of the care facility		
Strong management and leadership	19. Transformational leadership	Global Transformational Leadership (GTL) scale (1 to 5)
Person centered care / approach to people with dementia/ positive person work / insufficient attention to resident’s occupational needs, initiatives and capabilities / Lack of understanding importance of occupation / Task oriented working / Prioritization of physical over psychosocial needs / Organizational routines limiting autonomy of residents/ Family involved in assessment activities	20. Person-centered care	Person-centered Care Assessment Tool (P-CAT; 1 to 5). 12 of the 14 items were used based on factor analyses: items 4 and 13 were dropped
	21. Family perceived involvement	Family Perception of Caregiver Role (FPCR) instrument (1 to 7). 23 of the 31 items were used based on factor analyses: items 3–6, 8, 13, 14 and 17 were dropped.
Philosophy of care	22. Philosophy of care	Unanimity in care philosophy questionnaire (1 to 5)
Psychotropic / anticholinergic / antipsychotic / sedative drug use	23. Psychotropic drug use	Average number of prescribed psychotropic drugs per resident (0.17 to 2.38)
Use of physical restraints	24. Physical restraint use	Average number of physical restraints applied per resident (bed rails not included; 0 to 0.74)
Characteristics of job strain as perceived by care staff		
Perceived high workload / lack of time / emotional and task related demands / work stress	25. Job demands	Work and time pressure subscale of the Leiden Quality of Work Questionnaire (LQWQ; 1 to 4)
	26. Decision authority	Decision authority subscale of the LQWQ (1 to 4)
	27. Burn-out complaints	emotional exhaustion subscale Utrecht Burn-out Scale-C (UBOS; 0 to 6)
Work satisfaction	28. Job satisfaction	Job satisfaction subscale LQWQ (1 to 4)
Perceived support of staff	29. Social support colleagues	Social support co-workers subscales LQWQ (1 to 4)
Perceived support of manager	30. Social support supervisor	Social support supervisor subscales LQWQ (1 to 4)
Competence to provide suited activities / Perceived success in treatment of residents	31. Feelings of competence	Feelings of competence subscale UBOS (0 to 6)
Characteristics of the physical care environment		
Number of residents in facility / smaller size of facility	32. Total number of residents of care home	Total number of residents of the dementia care wards of care home (6 to 161)
Small scale living facility / Familiar environment / homelike environment or atmosphere / household environment / social interaction enhancing environment / small group of people	33. Group living home care characteristics	14-item version of the Questionnaire ‘Group Living Home Characteristics’ (0 to 56)
Characteristics of the organization of activities		
(Absence of) large group activity offer or standardized activities/ Individual and small group activity offer / lack of organized activities / activity choices / availability of various and ongoing or continuous activities / focus on everyday occupation	34. Central activity program	Are there activities organized by a central activity program, are there activities that are offered in the living rooms, and are there activities organized in the form of in clubs (more options are possible) – all variables dichotomized in yes or no.
	35. Activities that are offered in the living rooms	
	36. Activities in the form of in clubs	
Activity provision not restricted to activity workers / no specialized worker perspective	37. Activities are (also) offered by care staff	yes or no
Activities provided by recreational staff / absence of activity staff	38. Availability of recreational or activity staff	Number of available hours per week per resident of dementia care wards (0 to 4.71)
Family involvement (hr/week)	39. Availability of help of family caregivers	Number of hours of help from family caregivers per week per resident (0 to 4.20)
	40. Availability of help of volunteers	Number of hours of help from volunteers per week per resident (0 to 6.67)

In this Table, only predicting variables have been included that were measured in the LAD-study. In the literature, more predictors were suggested (see Additional file 1)

The characteristics age, gender, having a life partner, length of stay in the care home, ADL dependency, cognitive state and behavioral symptoms were assessed as potential predicting factors relating to residents. Based on the hypotheses that a recent transfer to a long-term-care home might positively or negatively influence activity involvement [9, 16], length of stay was dichotomized in shorter (<6 months) and longer length of stay (>6 months).

ADL dependency was measured with the Katz inventory [58] (Cronbach’s α = .918 in this sample; range 1–7). The score on this scale was treated as a continuous variable, with a higher score indicating more ADL dependency. To specifically study the influence of mobility on activity involvement, the item of being able to transfer was also studied separately. For this purpose, this item was dichotomized in ‘yes’ (transferring independently with or without instrumental aids), and ‘no’ (hardly or not being able to transfer independently. Behavioral symptoms were measured using the Neuropsychiatric Inventory [59, 60] (NPI-Q; Cronbach’s α = .743 in this sample; range 0–36). The total score on this scale was treated as a continuous variable, with a higher score indicating more behavioral symptoms. In the NPI-Q, the occurrence of symptoms of delusions, hallucinations, irritableness, eating disorders, sleeping disorders, disinhibition, euphoria, repetitive behavior, depression, apathy, agitation, and anxiety are measured. Because the latter four items were explicitly mentioned in the literature as influencing activity involvement, these were also separately studied. All four symptoms were dichotomized in ‘no’ if the behavior only seldom occurred, or not at all. If they occurred on a regular basis, they were classified as ‘yes’. Data on cognitive status were studied with the Cognitive Performance Scale [61] (CPS; Cronbach’s α = .814 in this sample; range 0–6). The score on the CPS was studied as a continuous variable, with a higher score indicating more cognitive impairment.

*2) Characteristics of financial resources, staff ratio and staff educational level*

Information on staff ratio and care staff educational level was derived by obtaining the actual working schedules used in care homes. The number of working hours per week per resident during day-time was calculated, including the working hours of possible recreational workers. Information on education level was derived by calculating the percentage of the total staff ratio in which staff with education level three or higher was working. In the Netherlands, a healthcare worker’s education level ranges from 1 to 5. In the Dutch education system, level 2 is equivalent to nursing assistant in the USA, level 3 to certified nursing assistant, and level 4 and 5 to registered nurse - all likewise in the USA.

The availability of (para)medics was measured as the total number of hours that (para)medics (e.g. the nursing home physician, psychologist, physiotherapist, occupational therapist, dietician) were available weekly for the dementia care units. Next, these hours were divided by the total number of residents in these units.

The average of 6 months of sick leave data concerning the dementia care units were used to indicate instability of staff and abnormalities in schedules concerning staff quantity or quality, as well as information on the number of staff vacancies per resident.

*3) Characteristics of modern vs. traditional care culture*

Transformational leadership, person-centered care, unity in care philosophy, psychotropic drug prescription and physical restraint use, and family’s perceived involvement in care were studied as indicators of a modern (psychosocial) or traditional (medical) care home culture.

Transformational leadership was measured with a Dutch translation of The Global Transformational Leadership scale [62] (GTL), that consists of 7 items and asks staff members questions on how charismatic, innovative, supportive, empowering, encouraging and challenging their direct manager is. The measure proved to have high reliability in our sample (Cronbach’s α = .955 in our sample, range 1–5).

A Dutch translation of the Person-Centered Care Assessment Tool [63] (P-CAT) was used to measure the extent to which the care staff and care home operate in a person-centered manner. It contains questions on whether residents’ individual needs are inventoried daily, whether they can participate in individualized activities, and whether there is a focus on creating a calm and homelike environment. The original instrument consisted of 14 items. Factor analysis revealed that 2 items had to be left out of analyses to form a reliable scale. The scale ranges from 1 to 5 and had a Cronbach’s Alpha of .806 in our sample. A higher score indicates more person-centered care.

To study whether or not care facilities operated strongly from a certain philosophy of care regarding living arrangements, we designed the Unity in Care Philosophy questionnaire [64]. This instrument consists of 7 items (Cronbach’s α = .916 in our sample) reflecting common philosophy of care statements. Care staff are asked to what extent there are differences in opinion or doubts in their team regarding several statements, for example: 1) Freedom of choice for residents; 2) communication with family caregivers; and 3) accepting differences between colleagues. The instrument ranges from 1 to 5, with a higher score indicating more consensus on care philosophy.

The number of psychotropic drugs were measured using standardized registrations of prescribed benzodiazepines and anti-psychotic drugs on the day prior to the visit of the research assistant for all residents of the care home. The registrations were filled in by the old age care physicians of the care home. With the information from the registration forms, the total number of psychotropic drugs in the care home was computed and divided by the number of residents to gain an average number of prescribed drugs per resident.

In addition, the average number of physical restraints was measured by collecting data on the number of residents for whom physical restraints – for example fixation belts, chairs with table top, and extra deep chairs – were prescribed (also registered by the old age care physician). The total number of restraints was computed and divided by the number of residents. Because we studied the relationship with day-time activity involvement, the use of bed rails (night-time use) was left out of the analyses.

Family perceived involvement in care decision-making was measured within the family sample participating in the LAD-study, with the use of the Family Perception of Caregiver Role instrument [65] (FPCR). Examples of items of this instrument are ‘I feel like an outsider in the care of my relative’, ‘It is clear that the staff have the real say about what care will be provided and how’, and ‘I feel like staff are there to help me provide the best possible care for my relative’. Factor analyses made it clear that eight of the original 23 items should be left out of the analyses for the internal consistency of the scale (range 1–7; Cronbach’s α = .895 in our sample). The scale measures the extent to which family feels supported and involved in decisions and procedures concerning the care for their relative. A higher score on the FPCR represents more involvement in care.

*4) Characteristics of job strain as perceived by care staff*

Staff’s job satisfaction, job characteristics from the Job-Demand-Control-Support model [66] and burnout characteristics were used as measures for staff perceptions of job strain.

The Leiden Quality of Work Questionnaire [67] (LQWQ) was used to measure job satisfaction and job characteristics. The total scale measures 11 job characteristics. For this study, the five subscales concerning the JDCS model are used: the Job Satisfaction subscale measuring job satisfaction and intention to leave (Cronbach’s α = .857 in our sample), the Work and Time Pressure subscale measuring job demands (Cronbach’s α = .747), the Decision Authority subscale measuring job control (Cronbach’s α = .709 in our sample), the Social Support from the Supervisor subscale (Cronbach’s α = .912 in our sample) and the Social Support from Co-workers subscale (Cronbach’s α = .835 in our sample) measuring social support. All job characteristics were measured on a four-point scale ranging from (1) “strongly disagree” to (4) “strongly agree”. Per subscale, the answers were added and means were calculated (range 1–4). A higher mean score represents a higher presence of the job characteristic.

Burnout complaints were measured with the Dutch version of the Maslach Burnout Inventory [68], the Utrecht Burnout Scale -C [69] (UBOS). The subscales of emotional exhaustion (Cronbach’s α = .880 in our sample), and personal accomplishment (Cronbach’s α = .777 in our sample) were used in this study. Both scales range from 0 to 6, where a higher score indicates more emotional exhaustion or more feelings of competence respectively.

*5) Characteristics of the physical care environment*

To obtain data about the size of the care home, the total number of residents of the dementia units of the care home was registered.

As a measure for the presence of a homelike environment, the number of group-living home care characteristics were studied. Data on this indicator were obtained by the Questionnaire ‘Group Living Home Characteristics’ [70]. A principal axis analysis showed one factor with relatively high loadings (>0.4) of 14 items (range: 0–56; Cronbach’s α = .857). Examples of items are: Living rooms have a homelike atmosphere; dinner is prepared in the kitchen of the living rooms; care staff do housekeeping; and residents can get out of bed whenever they want. The response-categories have a 5 point Lickert scale format. A higher score indicates more characteristics of group-living home care.

Since the Group Living Home Characteristics questionnaire and the average number of residents per common living room (place where residents usually stay during the day) were highly correlated (.629), the latter information was left out of the analyses.

*6) Characteristics of the organization of activities*

The ways in which activities were offered at the care homes were inventoried by asking the manager whether central activities were provided in fixed schedules, whether they were offered in the common living rooms of the residents, or whether activities were organized in the form of clubs, for which a particular group of residents is registered according to their personal preferences (for example the yoga or music club). The manager could choose multiple options.

We also asked whether these activity offers were provided by care staff, activity or recreational staff, volunteers or family. Again, one could choose multiple options. A dichotomous variable was made for the sole provision of activities by activity or recreational staff, and for activity provision by care staff or a combination of staff functions.

Furthermore, the number of hours per week of recreational workers or activity staff that worked for the care home were collected and divided by the number of residents at the total facility site.

Finally, data on availability of help from family caregivers and volunteers were collected. In the interview with the care manager, he or she was asked to estimate how many hours a week family caregivers and volunteers were present to actually perform care or activity tasks in the living arrangement. These numbers were divided by the number of residents in the care home.

### Analysis

To study the effect of family- and staff-related predictors of activity involvement of the individual residents, the mean scores of care staff and family caregiver variables for each care home were calculated and added to the residents of the particular care home. When there were less than 4 questionnaires filled out by staff members or family caregivers of a care home respectively, the staff- or family caregiver-related predictive values of that care home were excluded from analyses, in order to minimize unrepresentative mean scores for a care home. Missing values were estimated and replaced with multiple imputation.

Multilevel analyses were performed to correct for our clustered data [71]. MLwiN 2.21 software was used as statistical computer program. The outcome variable ‘time involved in activities’ was highly skewed to the left. Therefore, it was dichotomized into low involvement (3 h in 3 days or less – in other words, 1 h a day or less); and high involvement (more than 1 h a day). Backward stepwise logistic regression analyses were performed to analyze which factors predicted higher activity involvement, stepwise excluding variables with the smallest and non-significant relationship to the outcome variable (*p* < .05).

Our 40 potential predicting variables were entered blockwise in the regression model: we first studied only which resident characteristics predicted high activity involvement, then which characteristics of financial resources were predictors, and so on. Ultimately, all remaining significant predictors for each block were put together in one model to perform a final backward regression analysis, in order to determine their relative impact on activity involvement.

## Results

### Sample

A total of 1389 observational questionnaires on residents with dementia were filled out by care staff - a response rate of 89%. Eight hundred eighty eight family caregivers returned their questionnaires (a response rate of 52%). Complete data on activity involvement were available for 1218 residents with dementia (88% of the returned questionnaires) representing 139 care facilities. A total of 2160 questionnaires were distributed to staff, and 1145 care workers participated and met our criteria, resulting in a response of 53%.

### Characteristics of the participants

In Table 3, the characteristics of the residents, staff and care homes concerning the six groups of predictors that were studied are presented. To give insight into variations in resident and care characteristics between the five Dutch care settings that were represented in this study, the later columns of Table 3 show the participants’ characteristics across these settings.

**Table 3:** Background characteristics of participants

	Overall sample	Type 1 CH ^a^	Type 2 CH ^a^	Type 3 CH ^a^	Type 4 CH ^a^	Type 5 CH ^a^
Variables *(if applicable: range)*	M (SD) or n (%)	M (SD) or n (%)	M (SD) or n (%)	M (SD) or n (%)	M (SD) or n (%)	M (SD) or n (%)
**1. Resident characteristics**	*n* = 1218	*n* = 249	*n* = 268	*n* = 242	*n* = 236	*n* = 223
Age *(42–101)*	M 84.10 (SD 7.42)	M 82.77 (SD 8.51)	M 85.62 (SD 6.10)	M 83.65 (SD 7.49	M 84.19 (SD 6.41)	M 84.16 (SD 8.21)
Female residents	n 921 (% 75.7)	n 189 (% 75.9)	n 213 (% 79.5)	n 165 (% 68.2)	n 178 (% 75.4)	n 176 (% 78.9)
Residents with life partner	n 301 (% 24.7)	n 72 (% 28.9)	n 64 (% 23.9)	n 57 (% 23.6)	n 50 (% 21.2)	n 58 (% 26.0)
Residents with length of stay 6 months or more	n 1067 (% 87.6)	n 218 (% 87.5)	n 231 (% 85.8)	n 216 (% 89.2)	n 207 (% 87.7)	n 196 (% 87.9)
CPS overall score *(0–6)*	M 3.99 (SD 1.46)	M 4.11 (SD 1.46)	M 4.00 (SD 1.49)	M 4.14 (SD 1.44	M 3.80 (SD 1.49)	M 3.87 (SD 1.40)
Residents with mild to moderate cognitive impairment *(CPS 0–3)*	n 408 (% 33.6)	n 76 (% 30.5)	n 95 (% 35.4)	n 67 (% 27.7)	n 89 (% 37.7)	n 81 (% 36.3)
Residents moderate to severe cognitive impairment *(CPS 4)*	n 297 (% 24.4)	n 61 (% 24.5)	n 55 (% 20.5)	n 57 (% 23.6)	n 62 (% 26.3)	n 27.8 (% 27.8)
Residents with severe to very severe cognitive impairment *(CPS 5–6)*	n 513 (% 42.1)	n 112 (% 45.0)	n 118 (% 44.0)	n 118 (% 48.8)	n 85 (% 36.0)	n 35.9 (% 35.9)
NPIQ *(0–36)*	M 11.52 (SD 6.31)	M 11.50 (SD 6.87)	M 11.29 (SD 6.33)	M 11.75 (SD 6.09	M 11.45 (SD 6.19)	M 11.66 (SD 6.03)
Residents with agitation symptoms	n 329 (% 27.1)	n 73 (% 29.6)	n 80 (% 29.9)	n 66 (% 27.3)	n 60 (% 25.4)	n 50 (% 22.4)
Residents with depression symptoms	n 318 (% 26.1)	n 69 (% 27.5)	n 58 (% 21.6)	n 61 (% 25.3)	n 74 (% 31.4)	n 56 (% 25.1)
Residents with anxiety symptoms	n 262 (% 21.5)	n 47 (% 19.0)	n 60 (% 22.4)	n 52(% 21.5)	n 54 (% 22.9)	n 49 (% 22.0)
Residents with apathy symptoms	n 541 (% 44.5)	n 122 (% 49.0)	n 113 (% 42.2)	n 108 (% 44.6)	n 46.2 (% 46.2)	n 90 (% 40.4)
Katz ADL dependency *(1–7)*	M 5.35 (SD 1.65)	M 5.49 (SD 1.61)	M 5.51 (SD 1.56)	M 5.35 (SD 1.69	M 5.20 (SD 1.71)	M 5.19 (SD 1.67)
Residents without or low ADL dependency *(Katz 1–3)*	n 181 (% 14.9)	n 32 (% 12.9)	n 32(% 11.9)	n 40(% 16.5)	n 18.2(% 18.2)	n 34(% 15.2)
Residents dependent in various ADL domains *(Katz 4–6)*	n 627 (% 51.5)	n 125 (% 50.2)	n 139(% 51.9)	n 117(% 48.3)	n 50.4(% 50.4)	n 127(% 57.0)
Residents dependent in all ADL domains *(Katz = 7)*	n 410 (% 33.7)	n 92 (% 36.9)	n 97(% 36.2)	n 35.1(% 35.1)	n31.4(% 31.4)	n 62(% 27.8)
Residents independent in transferring (with or without aids)	n 651 (% 53.6)	n 125 (% 50.2)	135 (% 50.4)	133 (% 55.0)	129 (% 54.7)	94 (% 57.8)
Minutes involved in 17 listed activities during the past 3 days *(0–1125)*	M 152.49 (SD 166.80)	M 120.66 (SD 150.32)	M 143.53 (SD 156.71)	M 136.08 (SD 163.07)	M 191.03 (SD 177.60)	M 175.84 (SD 178.46)
Residents ≤3 h involved in activities during past 3 days	n 822 (% 67.5)	n 186 (% 74.7)	n 180 (% 67.3)	n 183 (% 75.6)	n 133 (% 56.4)	n 140 (% 62.8)
Residents >3 h involved in activities during past 3 days	n 396 (% 32.5)	n 63 (% 25.3)	n 88 (% 32.7)	n 59 (% 24.4)	n 103 (% 43.6)	n 83 (% 37.2)
**2. Characteristics of resources of finances, staff ratio and educational level**	*n* = 139	*n* = 28	*n* = 30	*n* = 28	*n* = 28	*n* = 25
Staff ratio *(13.86–30.48)*	M 20.86 (SD 3.61)	M 18.89 (SD 3.46)	M 20.49 (SD 3.57)	M 21.55 (SD 3.52)	M 21.59 (SD 3.24)	M 21.92 (SD 3.69)
Staff with education level 3 or higher *(22.70–100)*	M 63.55 (SD 15.58)	M 58.27 (SD 13.83)	M 63.58 (SD 13.65)	M 67.49 (SD 18.76)	M 61.85 (SD 12.69)	M 66.97 (SD 17.67)
Average % of sickness leave *(0.82–17.0)*	M 6.22 (SD 3.09)	M 7.10 (SD 2.00)	M 5.12 (SD 2.60)	M 5.96 (SD 2.62)	M 6.22 (SD 3.49)	M 6.86 (SD 3.51)
Number of vacancies per resident *(0–0.22)*	M 0.016 (SD 0.034)	M 0.015 (SD 0.023)	M 0.013 (SD 0.028)	M 0.022 (SD 0.036)	M 0.022 (SD 0.052)	M 0.008 (SD 0.022)
Hours / week (para) medics per resident *(0–5.06)*	M 1.32 (SD 0.93)	M 1.70 (SD 1.11)	M 1.24 (SD 0.92)	M 1.38 (SD 0.88)	M 0.91 (SD 0.55)	M 1.38 (SD 0.99)
**3. Characteristics of modern or traditional care culture of the care facility**	*n* = 139	*n* = 28	*n* = 30	*n* = 28	*n* = 28	*n* = 25
GTL *(1.55–4.81)*	M 3.23 (SD 0.63)	M 3.13 (SD 0.67)	M 3.10 (SD 0.69)	M 3.29 (SD 0.65)	M 3.35 (SD 0.62)	M 3.30 (SD 0.54)
P-CAT *(2.76–4.35)*	M 3.62 (SD 0.34)	M 3.36 (SD 0.33)	M 3.50 (SD 0.24)	M 3.73 (SD 0.26)	M 3.87 (SD 0.31)	M 3.62 (SD 0.31)
Unanimity in care philosophy *(2.39–4.57)*	M 3.38 (SD 0.39)	M 3.19 (SD 0.33)	M 3.28 (SD 0.37)	M 3.53 (SD 0.32)	M 3.54 (SD 0.42)	M 3.35 (SD 0.37)
FPCR ( *3.98–6.47)*	M 5.51 (SD 0.46)	M 5.31 (SD 0.42)	M 5.38 (SD 0.45)	M 5.50 (SD 0.44)	M 5.74 (SD 0.39)	M 5.66 (SD 0.43)
Number of psychotropic drugs per resident *(0.17–2.38)*	M 0.90 (SD 0.36)	M 1.08 (SD 0.41)	M 0.84 (SD 0.33)	M 0.92 (SD 0.29)	M 0.74 (SD 0.36)	M 0.95 (SD 0.35)
Number of physical restraints per resident *(0–0.74)*	M 0.11 (SD 0.13)	M 0.17 (SD 0.14)	M 0.12 (SD 0.18)	M 0.09 (SD 0.08)	M 0.09 (SD 0.12)	M 0.11 (SD 0.11)
**4. Characteristics of workload as perceived by care staff (mean scores per care home)**	*n* = 139	*n* = 28	*n* = 30	*n* = 28	*n* = 28	*n* = 25
LWQ Job satisfaction *(2.28–3.75)*	M 3.04 (SD 0.26)	M 2.85 (SD 0.24)	M 3.04 (SD 0.22)	M 3.09 (SD 0.25)	M 3.20 (SD 0.25)	M 3.04 (SD 0.25)
LWQ Job demands *(1.70–3.20)*	M 2.45 (SD 0.29)	M 2.70 (SD 0.25)	M 2.50 (SD 0.19)	M 2.41 (SD 0.27)	M 2.22 (SD 0.25)	M 2.41 (SD 0.27)
LWQ Autonomy *(2.33–4.00)*	M 2.95 (SD 0.21)	M 2.82 (SD 0.21)	M 2.88 (SD 0.14)	M 2.98 (SD 0.17)	M 3.11 (SD 0.16)	M 2.98 (SD 0.25)
LWQ Social support manager *(2.17–3.68)*	M 3.04 (SD 0.30)	M 2.97 (SD 0.35)	M 3.00 (SD 0.30)	M 3.06 (SD 0.34)	M 3.08 (SD 0.26)	M 3.09 (SD 0.26)
LWQ Social support coworkers *(2.40–3.88)*	M 3.21 (SD 0.23)	M 3.15 (SD 0.23)	M 3.20 (SD 0.21)	M 3.25 (SD 0.20)	M 3.22 (SD 0.28)	M 3.20 (SD 0.48)
UBOS emotional exhaustion *(0.61–3.46)*	M 1.76 (SD 0.52)	M 2.08 (SD 0.49)	M 1.82 (SD 0.57)	M 1.74 (SD 0.45)	M 1.47 (SD 0.41)	M 1.68 (SD 0.48)
UBOS Burnout personal competence ( *3.71–5.79)*	M 4.73 (SD 0.31)	M 4.53 (SD 0.30)	M 4.72 (SD 0.32)	M 4.76 (SD 0.25)	M 4.84 (SD 0.29)	M 4.82 (SD 0.33)
**5. Characteristics of the physical care environment**	*n* = 139	*n* = 28	*n* = 30	*n* = 28	*n* = 28	*n* = 25
Number of residents in facility *(6–161)*	M 40.43 (SD 32.44)	M 69.54 (SD 38.68)	M 23.37 (SD 8.63)	M 60.61 (SD 34.03)	M 20.71 (SD 15.49)	M 27.80 (SD 16.14)
Number of residents per living room *(5–28)*	M 9.26 (SD 3.88)	M 12.18 (SD 2.99)	M 12.09 (SD 4.84)	M 8.07 (SD 2.91)	M 6.41 (SD 0.87)	M 7.12 (SD 1.37)
Group living home care characteristics *(9–51)*	M 31.32 (SD 10.59)	M 22.58 (SD 7.29)	M 22.47 (SD 5.74)	M 33.64 (SD 8.04)	M 42.04 (SD 5.48)	M 37.12 (SD 8.77)
**6. Characteristics of the organization of activities**	*n* = 139	*n* = 28	*n* = 30	*n* = 28	*n* = 28	*n* = 25
Care homes with activities arranged in clubs	75 (% 54.0)	n 21 (% 75.0)	n 11 (% 36.7)	n 19 (% 67.9)	n 9 (% 32.1)	n 15 (% 60.0)
Care homes with central activity program	119 (% 85.6)	n 25 (% 89.3)	n 25 (% 83.3)	n 26 (% 92.9)	n 23 (% 82.1)	n 20 (% 80.0)
Care homes with activities in living room	132 (% 95.0)	n 28 (% 100)	n 28 (% 93.3)	n 27 (% 96.4)	n 27 (% 96.4)	n 22 (% 88.0)
Care homes where activities are (also) offered by care staff	124 (% 89.2)	n 24 (% 85.7)	n 26 (% 86.7)	n 27 (% 96.4)	n 24 (% 85.7)	n 23 (% 92.0)
Hours / week recreational staff per resident *(0–4.71)*	M 0.78 (SD 0.78)	M 1.12 (SD 0.85)	M 1.17 (SD 0.92)	M 0.75 (SD 0.50	M 0.12 (SD 0.19)	M 0.74 (SD 0.69)
Hours of help from family caregivers per resident per week *(0–4.20)*	M 0.36 (SD 0.68)	M 0.24 (SD 0.53)	M 0.16 (SD 0.28)	M 0.33 (SD 0.67	M 0.66 (SD 0.81)	M 0.39 (SD 0.90)
Hours of help from volunteers per resident per week *(0–6.67)*	M 1.06 (SD 1.08)	M 0.62 (SD 0.78)	M 0.89 (SD 0.57)	M 1.21 (SD 1.25	M 1.58 (SD 1.46)	M 0.95 (SD 0.93)

^a^ Types of care homes that were represented in the LAD-study: 1) traditional large scale nursing homes; 2) nursing home wards in homes for the aged; 3) large scale group living homes (>36 residents with dementia); 4) small scale group living homes (< 36 residents) only providing group living home care, 5) small scale group living homes with other care forms at same location

*CPS* cognitive performance scale, *NPI-Q* 12 item Neuropsychiatric Inventory questionnaire; KATZ, ADL dependency; UBOS, burnout questionnaire; LQWQ, Leiden Quality of Work Questionnaire; Staff ratio in hours of care staff per resident per week; GTL, transformational leadership; P-CAT, person-centered care; FPCR, Family Perception of Caregiver Role, Group living home characteristics, short version of the Questionnaire ‘Group Living Home Characteristics’

#### Residents’ characteristics

Overall, residents had a mean age of 84. The majority of the sample was female (75%), and 25% had a life partner. Most residents resided longer than 6 months in the care home (88%). With regard to their stage of dementia, 34% of the residents had mild to moderate dementia; 24% had moderate to severe dementia, and 42% had severe to very severe dementia. The average resident had some behavioral symptoms. Agitation, depression and anxiety were present in 22% to 27% of the sample, and 45% had apathy symptoms. The average resident needed help in most domains of Activities of Daily Living. Only 15.4% needed help in less than three ADL domains. The activity involvement of the residents greatly varied. On average, residents were involved in the 17 listed activities for 152 min during 3 days. Of all residents, 32.5% were involved in activities for 1 h a day or more.

#### Staff ratio and educational level

On average, the participating care homes had a staff ratio of 21 h a week per resident, and 64% of the scheduled staff had an educational level of 3 or higher. Care homes had an average sick leave number of 6%, meaning that 6% of the originally scheduled care staff were absent from work due to sickness and had to be replaced. Care homes had around .02 vacancies per resident on average. Per week, (para)medics were involved for somewhat more than 1 h per resident. There was considerable variation in most of these organizational characteristics however: staff ratio ranged from almost 14 to 30 h per resident per week, and the percentage of staff with a higher educational level ranged from 23% to 100%. The average percentage of sickness leave ranged from less than 1% to 17%.

#### Traditional vs. modern care culture

Concerning care culture, facilities scored moderately high on transformational leadership, person-centered care, unity in care philosophy (scores of 3 to 4 on a scale from 1 to 5), and family perceived involvement in care decision-making (score of 5.5 on a scale from 1 to 7). On average, 0.9 psychotropic drugs per resident were prescribed, and for 10% of the residents, a physical restraint was used. There was a large range however concerning these latter measurements.

#### Job strain as perceived by staff

Staff that contributed to this study had a mean age of 43 years and were predominantly female. On average, they were satisfied with their work, and experienced autonomy as well as social support from colleagues and their supervisor on a regular basis (scores of 3 on the 1–4 Lickert scale). They experienced moderate levels of work demands (score of 2.45 on the scale from 1 to 4). Concerning burnout-complaints, they experienced some emotional exhaustion and moderate to high levels of personal competence on average (score of 1.75 and 4.73 respectively on a scale from 0 to 6).

#### Physical care environment

Consistent with the study design, living arrangements varied greatly in size and in terms of group-living home characteristics. The total number of residents in the dementia units of the care homes ranged from 6 to 161 residents. Traditional large-scale care homes (type 1 as presented in Table 3) and large-scale group living home facilities (type 3) had the highest total numbers of residents, with average numbers of 70 and 61 residents respectively. The small-scale group living home facilities (type 4 and 5), as well as the traditional dementia care units in homes for the aged, had an average resident number of 21 to 28.

In the traditional types of nursing homes (type 1 and 2), residents lived together in groups of approximately 12, whereas the group-living home care facilities (type 3, 4, 5) had 6 to 8 residents per living room.

In the ‘archetypal’ small scale group living home facilities (type 4), the most characteristics of group living home care were present (on average, 42 out of the maximum score of 56). The alternative group living home care facilities (type 3 and 5) had a somewhat lower score on the Group Living Home Characteristics questionnaire (34 and 37 resp.). And the traditional nursing home care facilities had the fewest characteristics of group-living home care (type 1 had an average score of 23, and type 2 of 22).

#### Organization of activities

Almost all care homes (95%) offered activities in the common living rooms, and 86% provided activities in a central activity program. In 54% of the care homes, activities were arranged in the form of clubs. In 10% of the care homes, activities were only provided by activity or recreational staff, sometimes with help of volunteers or family. In the other care homes, activities were (also) provided by care staff. Structural help from family caregivers was much lower than help from volunteers (as estimated by the care manager 0.4 and 1 h a week on average per resident, respectively) and varied greatly between care homes.

### Results of blockwise analyses of predictors of activity involvement

Table 4 shows the results of the blockwise prediction analyses.

**Table 4:** Results of blockwise backward prediction analyses

	Higher activity involvement		
	*B*	*SE*	*OR*
*Block 1: characteristics of residents with dementia*			
Agitation	−.715***	.161	.489
Katz	−.212***	.047	.809
CPS	−.293***	.055	.746
*Block 2: characteristics of resources of finances, staff ratio and educational level*			
Staff ratio	.040*	.017	1.492
Education level	.009*	.004	1.009
*Block 3: characteristics of modern or traditional care culture*			
GTL	−.286*	.104	.751
FPCR	.315*	.141	1.370
Unity in Care Philosophy questionnaire	.484*	.170	1.623
*Block 4: characteristics of job strain as perceived by staff*			
LQWQ Working demands	−1.100***	.247	.333
LQWQ Social support supervisor	−.784***	.232	.457
*Block 5: characteristics of physical care environment*			
Total # of residents	−.008***	.002	.992
Group living home characteristics	.015*	.006	1.015
*Block 6: characteristics of organization of activities*			
Activities in clubs	−.281*	.123	.755
Help of volunteers	.125*	.056	1.142

* *p* < 0.05. ** *p* < 0.01, *** *p* < 0.001

CPS, cognitive performance scale; NPI-Q, 12 item Neuropsychiatric Inventory questionnaire; KATZ, ADL dependency; LQWQ, Leiden Quality of Work Questionnaire; Staff ratio in hours of care staff per resident per week; GTL, transformational leadership; P-CAT, person-centered care; Group living home characteristics, short version of the Questionnaire ‘Group Living Home Characteristics

#### Resident characteristics

The prediction model of resident characteristics was filled with the variables age, gender, having a life partner, length of stay, ADL dependency, immobility, cognitive impairment, overall behavioral symptoms, and depression, agitation, anxiety, and apathy. Backward regression analysis revealed that out of these variables only agitated behavior (Odds Ratio .489), ADL dependency (OR .809) and cognitive impairment (OR .746) were predictors of activity involvement. As shown by the Odds Ratios, these characteristics were all negatively related to higher activity involvement.

#### Resources of finances, staff ratio and educational level

Concerning the financial resources of the care homes, staffing levels, staff educational level, availability of (para)medics, the number of vacancies and sick leave were entered in the model. It was found that a higher staff ratio (OR 1.492) and a higher percentage of staff with educational level three or higher (OR 1.009) predicted higher activity involvement of residents.

#### Traditional vs. modern care culture

Out of the variables representing care culture - transformational leadership, person-centered care, family perceived involvement, unity in the philosophy of care, psychotropic drug prescription and physical restraint use - three variables had a predictive value. Higher scores for family perceived involvement (OR 1.37) and more unity in care philosophy (OR 1.623), predicted higher activity involvement; whereas more transformational leadership predicted lower activity involvement by residents (OR .751).

#### Job strain as perceived by staff

Our analysis of the variables that represented job strain factors commenced with the variables job demands, decision authority, burn-out complaints, job satisfaction, social support from the supervisor, social support of colleagues, and feelings of competence. The results show that higher job demands (OR .333) were related to lower activity involvement, as was as more perceived social support from the supervisor (OR .457).

#### Physical care environment

With regard to characteristics of the physical environment, the number of residents in the dementia care units, and group living home characteristics were entered as variables. Both factors predicted activity involvement: more residents in the dementia care units (OR .992) was related to less activity involvement, whereas more group living home characteristics predicted higher activity involvement (OR 1.015).

#### Organization of activities

When looking at the way in which activities are organized in care homes with the variables of a central activity program, activities organized in clubs, activities offered in the living room, activities also organized by care staff, availability of recreational staff, and hours of informal help, two variables are found to have a predictive value. More help of volunteers (OR 1.14) predicted higher activity involvement, while an activity offer organized in activity clubs was related to lower activity involvement (OR .755).

### Results of the end model of predictors of activity involvement

In Table 5, the end model is presented after putting all significant predictors of the different blocks in one prediction model (agitation, ADL dependency, cognitive impairment, staff ratio, education level, transformational leadership, family perceived involvement, unity in care philosophy, job demands, social support supervisor, total number of residents in dementia units, group living home characteristics, activities organized in clubs, and availability of help from volunteers). Out of the initial 40 factors that were studied, seven variables were found to be significant predictors in the end model, and thus played a key role in the activity involvement of residents with dementia. Agitated behavior (OR .490), more ADL dependency (OR .805) and more cognitive impairment (OR .733) were negatively related to activity involvement. A higher staff educational level (OR 1.012) predicted higher activity involvement, whereas more perceived job demands among staff (OR .435) and higher levels of perceived supervisor support (OR .458) negatively influenced activity involvement. Furthermore, a higher total number of residents in the dementia care units (OR .994) was related to less activity involvement.

**Table 5:** End results when all predictive factors of blockwise analyses are put together in 1 model

	Higher activity involvement		
	*B*	*SE*	*OR*
Agitation	−.713***	0.163	.490
Katz	−.217***	0.048	.805
CPS	−.293***	.056	.746
Education level staff	.012**	.004	1.012
LWQ Working demands	−.833**	.271	.435
LWQ Social supervisor support	−.822**	.251	.440
Number of residents at facility site	−.006**	.002	.994

* *p* < 0.05. ** *p* < 0.01, *** *p* < 0.001

CPS, cognitive performance scale; NPI-Q, 12 item Neuropsychiatric Inventory questionnaire; KATZ, ADL dependency; LQWQ, Leiden Quality of Work Questionnaire

## Discussion

In this study, a wide range of variables that were previously found or thought to impact the activity involvement of long-term-care home residents with dementia were studied. We found that several factors significantly predicted higher activity involvement - defined as involvement in activities for more than 1 h a day. These factors were a higher staff ratio and higher staff educational level, more involvement of family caregivers in the decisions and procedures in the care for their relative, greater unity in the care philosophy of staff, more group living home care characteristics, and more help from volunteers at the care home. Agitated behavior, cognitive impairment and ADL dependency were negatively associated with higher activity involvement, as was transformational leadership, more perceived job demands and more supervisor support, more residents in the care home and offering activities in the form of clubs. Of these predictors, the presence of agitated behavior in residents, physical and cognitive functional level, more staff with educational level 3 or higher, more perceived job demands and social supervisor support, and the total number of residents in the care home were found to have the most important impact on activity involvement.

The finding that more cognitively and physically impaired residents are less involved in activities is consistent with the literature [9, 15–17, 19, 20, 22, 24, 26]. Although activity involvement remains important for people with more cognitive and physical limitations [14], it seems difficult for staff to reach high levels of occupation among these residents.

This might be explained by the time pressure on care staff ensuing from complex care demands. Presumably, more physically and cognitively impaired residents need more time-consuming physical care, leaving care staff with less time to offer activities and forcing them to mainly focus on care instead of recreational tasks. If this is true, care staff must learn to integrate physical care with meaningful occupation when residents’ care demands increase, in order to address higher activity and wellbeing levels amongst more impaired residents. Examples include singing, playing someone’s favorite music, or giving a massage while bathing. It is about making contact, and taking time to do so [72].

On the other hand, the negative relationship between more cognitive and physical impairment and activity involvement can be caused by limited knowledge among staff on how to offer appropriate activities to this resident group. Engaging severely impaired residents in activities requires special skills and the use of adjusted materials, based on the (limited) capabilities that are preserved [73]. With the increasing care dependency of residents in long-term-care homes, it is important to train care staff in assessing the capabilities and interests of residents and developing the required activity skills [48, 72, 73], also for the involvement of more care-dependent residents.

The same holds for our study finding that residents with agitated behavior are less likely to be involved in activities. If activities are tailored to the specific level of function, residents with this behavior might still be able to be engaged [18].

Educating staff in the provision of suitable activities and the integration in the daily care thus seems a key factor for increasing activity involvement of residents with dementia. In the Netherlands, some care homes are working already with ‘recreational coaches’: former recreational staff that are tasked with developing individual activity plans for residents, and teaching regular care staff (with a nursing education) to integrate the provision of activities into their daily work. Although individual activity plans may suit residents better than the traditional organization of activities [4], the recreational coach is also often the result of a financial reorganization, whereby the team of recreational workers that were responsible for all activity involvement of residents, is limited to one or two staff members that are labeled as ‘recreational coaches’. The level of knowledge of the therapeutic value of activities for residents (e.g. gaining self-esteem, social contact, activation, stimulation of the senses or memory, emotional expression), and of the available materials and activity types (e.g. reminiscence activities, sports and exercise materials for older people, material for sensory stimulation) is often low, as is sometimes the willingness among care staff to perform activities. This reorganization of activity provision therefore seems to have had a negative instead of positive effect on activity involvement among residents. The limited attention to activities by care homes and the need for the development of skills amongst care staff has recently been recognized by the Dutch government. Care homes receive substantial fees when they measurably stimulate activity provision amongst their care staff, during the period from 2016 to 2020 [74]. Hopefully, this will lead to the development of sustainable knowledge and skills among care staff on this topic. This movement can be strengthened by including activity provision in the training for RNs or CNAs.

Although offering activities to residents that are adjusted to their competences and interests is important [72], the development in care homes to organize activities in clubs for fixed small groups of residents based on their interests and life history, did not prove to be beneficial in this study. This finding might be explained by a deprivation of activity involvement in daily life outside the club offer, which is often only provided once a week. Although certain types of activities are known to particularly influence wellbeing [46, 49], it seems that frequent activity involvement is more important than involvement in a specific activity sporadically [75]. It may be also be the case that residents who do not thrive in group activities are overlooked with a club-wise activity arrangement.

Concerning the environment, a smaller number of residents at the total facility site was shown to be an important predictor of activity involvement of residents, as was also found in previous research [44]. Consistent with the literature, group living home care characteristics were likewise found to be positively related to higher activity involvement of residents with dementia. Ideally, in group living care facilities, small teams of staff provide care to a small group of residents, enabling staff to get to know residents better [76]. The homelike environment that invites residents to participate in household chores and normal life is assumed to result in higher activity involvement [44, 77]. Although the concept of group-living home care was introduced years ago (the first Dutch small-scale group living home facility dates back to 1989), and its principles are widely recognized as good dementia care practice [77], some care facilities still struggle to capture the essence of the concept and to put the accompanying working style into practice. For example, there are modern group living home care facilities in the Netherlands with large kitchens in each living area to cook with or for residents, but where the value of cooking meals is not recognized and the kitchens are not used [78]. However, the extent to which group-living home care is provided, was found to be subordinate to the number of residents at the total facility site, when both factors were added to the end model of predictors. This is an important finding, since many care facilities try to offer group living home care to larger clusters of resident groups. Providing small-scale care within a large- scale setting, might not be a good alternative for the archetypal [76] small-scale group living home care in terms of residents’ occupation. Perhaps, providing care on a large scale hampers the care home in providing truly individually tailored care, based on personal contact with residents and family caregivers. We did find that the level of involvement of relatives in decision-making about the care that is delivered, and better communication with relatives and staff, also predicted higher activity involvement, although this factor was of secondary importance.

This might be explained by the finding that meaningful occupation is seen as an important aspect of quality of care by family caregivers [5, 6], and that their involvement leads to better advocacy of the provision of activities to their relative with dementia. Or that family involvement leads to more interaction and enthusiasm in staff to involve the person with dementia in activities.

Furthermore, a care philosophy that is clear among the care staff, for example on communication with family caregivers, also plays a role in higher activity involvement. In previous research, a clear care philosophy for staff, management as well as family, was found to be the key factor in providing good care for living arrangements for people with dementia because it served as a true guide for how to deliver care, and it provided answers in difficult situations [79].

Inconsistently with the literature, the supervisor support perceived by care staff was found to be negatively related to higher activity involvement. Based on the assumption that care staff would find themselves more supported in the choices they make, for example in spending time on interacting with residents, we did not expect to find a negative association. The same holds for transformational leadership, since it stands for being a role model, being supportive, giving room for the own creativity and ideas of staff, and being visionary [80]. If a supervisor is not activity-minded however, supportive leadership might result in less activation than directive leadership by someone who strongly values activity provision to residents. More research is needed to explore this relationship.

In our study we found that higher job demands as perceived by care staff strongly predicted residents’ lower activity involvement. Several interventions to reduce job demands and job strain are proposed. These include giving staff more influence in making their own work schedules [81], giving them more decision-making authority [82], reviewing time-consuming rules and regulations which care staff have to act upon (the Dutch government is currently working on this [81]), or replacing working routines with the provision of person-centered care [83]. However, it is likely that job demands were at least partly caused by staffing levels, since these factors were correlated in this study.

Both higher staff ratio (subordinate) and higher staff educational level predicted greater activity involvement among residents. These factors are based on the financial resources of care facilities and are often perceived as hard to influence in times of economic recession. However, the care homes in our study varied greatly in educational level and staff ratio (ranging from 23 to 100% of staff with educational level 3 or higher, and 13.86 to 30.48 h of care staff a week per resident respectively) while having more or less the same financial input per resident. Staff ratio and educational level were not correlated in the analyses. In other words, a higher staffing level was not explained by a higher percentage of staff with a lower educational level. This insight highlights the urgent need for care homes to look into the distribution of their financial means. A less hierarchical organization of the larger care providers and a review of overhead costs (for example, losing the secretary, policy makers or the laundry service), might be a key factor in better spending the available means.

Attracting volunteers can be another solution to increase the number of people who are willing to engage residents in activities. In this study, we found that more hours of help from volunteers related to higher activity involvement among residents. Earlier, we found that there is much variety in the number and quality of volunteers available in care homes [79]. Several care homes have reported that they experience difficulty in finding and retaining volunteers. Care homes with many volunteers report that it requires creativity and reciprocity to attract and keep volunteers. A culture change is needed: from care homes where volunteers are perceived as visitors who solely contribute to the organization, to a place where they are truly part of the organization, where they feel welcome and where they contribute but also gain from their work, such as receiving learning possibilities, experience being part of a team, or receive support in a job-finding process. Care homes may be helped by sharing experiences. This requires the willingness to do so, which is not always the case when policy is focused on market mechanisms, giving care homes the idea that they need to compete.

To summarize, a change towards better education on activity provision and more archetypal small- scale group living home care provision, with a clear wellbeing-focused philosophy of care among staff and management and good cooperation with family in care provision for a resident, might result in higher activity involvement. However, appropriate conditions for good care provision need to be created first.

This study has strengths and limitations. Strong features are the large numbers of participants, representing a large number of dementia care homes in the Netherlands, and the wide variety of factors included in this study. A limitation is that, although causality is an underlying assumption in backward prediction analyses, this study gives no causal certainty over the relationships found due to the cross-sectional character of our study.

Furthermore, we were not able to study all factors that were found or proposed in literature to influence activity involvement, since not all factors were measured in the LAD-study. Therefore, some important predictors might have been missed, for example the role of specific environmental features (e.g. access to a garden [34] or light intensity in the living arrangement [23, 47]). For most factors that were studied, there was a clear measurement instrument available in the LAD-study. However, sometimes, an instrument was used that approached a factor mentioned in literature (e.g. for the factor ‘instability in care teams’ we used the number of vacancies per resident, and for *‘knowledge about dementia/lack of skills/formal staff training’,* we used the percentage of staff with a higher education level).

For feasibility reasons, we were not able to train staff in completing the observational questionnaires on resident characteristics and outcomes. This may have influenced the data, since instruments were used that might have needed further explanation. To illustrate, about 1% of our study sample had a Cognitive Performance Scale (CPS) sore of 0, indicating that these persons had no signs of cognitive problems. Since all residents participating in this study lived in dementia care units based on a diagnosis of dementia or other cognitive problems, a score of 0 is questionable. On occasion, a person might have scored low on the CPS due to an alternative form of dementia without clear signs of memory impairment in the earlier stages of the disease (for example frontal lobe dementia). But when talking with staff about their scorings of, for example, the CPS, we found that some of them overestimated the cognitive performances of residents based on their own support. For example, they scored a resident who suffered from severe aphasia as ‘having no problems in making himself clear’, because they normally could understand the person without using many words. Based on this experience, we strongly recommend clear instructions for care staff on filling in the CPS before use.

Moreover, the reliability and validity of the instrument used for measuring time of activity involvement is unclear. To our knowledge, little specific information is available on the intra- and interrater reliability of the standardized Activity Pursuit Patterns of the MDS [56, 57]. It is mentioned however that the instrument was filled in with little accuracy [84]. Our experience with the instrument confirms this. The instrument relies on the observations of care staff regarding activity involvement by residents during the past 3 days. It is doubtful that observations could be recorded entirely by the staff member filling in the questionnaire, all the more so because in this study, the Activity Pursuit Patterns was expanded with a time variable. Care staff work in shifts, making them dependent on reports and observations of colleagues. This probably resulted in estimations of time involved in activities instead of real-time involvement. Also, the fact that residents were reported to be involved in certain types of activities for extreme lengths of time, makes it likely that some staff found it difficult to discriminate actual activity involvement from being present in a room with stimuli. This could have resulted in an overestimation of activity involvement. For this reason, the activities talking, watching television and listening to music or radio, were excluded from the analyses, with consequences for the reliability of the data. Unfortunately, at the time of data collection, no good alternative instrument was available in terms of psychometric properties and feasibility. Ideally, activity involvement is measured by real-time observational instruments such as Dementia Care Mapping [13]. However, Dementia Care Mapping is time-consuming, and it requires certified ‘mappers’ to collect the data. Furthermore, occupation of residents is only measured when they are in the common living room of their care unit, thereby eliminating the activities that are done outside this common space, for example in recreational areas or the private room of the person with dementia. Just recently, the Maastricht Electronical Daily Life Observation (MEDLO) method was developed [85]. With MEDLO, occupation in daily life of residents with dementia throughout the care home is observed using tablets, making it more easy to report on. However, a convenient sample of residents available at that time is observed and trained observers still have to be present at the location sites. For a large-scale study such as the LAD-study, staff observations of actual activity involvement are preferable. Staff have to be trained properly though, mainly in differentiating actual involvement in activities from being present in a room where activities are available.

## Conclusions

The lack of activity involvement by care home residents with dementia may be targeted with programs focusing on: 1) Reducing the working demands that are experienced by care staff; 2) Increasing staff’s educational level and staffing levels; 3) Training staff in providing suitable and accessible activities concerning the behavior, cognitive capacity and functional ability of residents and the integration of activities in daily care; and 4) Limiting resident numbers at a facility site and securing the proper implementation of the essence of the group living home care environment; furthermore, 5) Working by a clear philosophy on care that focuses on the wellbeing of residents and the involvement of family caregivers, and 6) Attracting and retaining volunteers might help increase activity involvement amongst residents with dementia.

To act upon these directions, the redistribution of the available means within care homes might be essential. Therefore, the key factor in turning around passiveness may still be recognizing the value of activities for residents with dementia, both by care staff, care home directors and policy makers.

## Additional files

Additional file 1: Literature review on predictors of activity involvement. Overview of enabling and disabling factors of activity involvement of residents in long term dementia care as studied or suggested in literature (using search string ‘[dementia OR alzheimer’s] AND [long term care OR nursing home OR elderly care OR homes for the aged] AND [activity OR occupation OR leisure OR meaningful activities OR engagement OR involvement OR wellbeing OR quality of life]’ in pubmed, web of science and psychinfo, following snowball method) [86, 87]. (DOCX 29 kb)

Additional file 2: Family caregiver questionnaire. Measures and operationalization of standardized questionnaire for family caregivers in the second measurement cycle of the LAD-study [88, 89]. (DOCX 12 kb)
