# Supplementary material for: Predictors of activity involvement in dementia care homes: a cross-sectional study
Source: BMC Geriatr. 2017 Aug 4;17:175. doi: 10.1186/s12877-017-0564-7 (PMC5545000; doi:10.1186/s12877-017-0564-7)
Supplement: Supplementary file 1 — Literature review on predictors of activity involvement. Overview of enabling and disabling factors of activity involvement of residents in long term dementia care as studied or suggested in literature (using search string ‘[dementia OR alzheimer’s] AND [long term care OR nursing home OR elderly care OR homes for the aged] AND [activity OR occupation OR leisure OR meaningful activities OR engagement OR involvement OR wellbeing OR quality of life]’ in pubmed, web of science and psychinfo, following snowball method) [86, 87]. (DOCX 29 kb) [file 12877_2017_564_MOESM1_ESM.docx]

**Literature review on the predictors of activity involvement**: overview of enabling and disabling factors of activity involvement of residents in long term dementia care as studied or suggested in literature (using search string ‘[dementia OR alzheimer’s] AND [long term care OR nursing home OR elderly care OR homes for the aged] AND [activity OR occupation OR leisure OR meaningful activities OR engagement OR involvement OR wellbeing OR quality of life]’ in pubmed, web of science and psychinfo, following snowball method)

| **Authors / year** | **Study description** | **Type of study** | **# study subjects** | **Factor(s)** | **Enabling / disabling** | **Suggested / studied in quantitative research / study result from qualitative research** |
| --- | --- | --- | --- | --- | --- | --- |
| Abrahamson *et al.*, 2012^19^ | Study on the influence of cognitive impairment and facility characteristics on quality of life | Cross sectional study | 13983 | RESIDENTS  Cognitive impairment  PHYSICAL ENVIRONMENT  Special care unit | Disabling  Enabling | Studied, confirmed  Studied, confirmed |
| Bates-Jensen, Schnelle et al., 2004^30^ | To study the effect of low staffing levels on daytime sleep, social engagement and food and fluid intake | Cross sectional study | 882 | STAFF RATIO  Low staffing level | disabling | Studied, confirmed |
| Van Beek *et al.*, 2010^26^ | Study on explanatory role of residents’ characteristics and differences in care setting on social engagement including individual activities | Cross sectional study | 502 | RESIDENTS  Higher age  More social resources  Problems in ADL  Problems in cognitive performance | Disabling  Enabling  Disabling  Disabling | Studied, n.s.  Suggested  Studied, confirmed  Studied, confirmed |
| Buettner, 1999^29^ | Study on the effects of handmade recreational items on behavior of residents and impact on family visits, staff knowledge and volunteer involvement | Experimental study with cross over design | 55 | STAFF RATIO  Shortness of time and resources  ACTIVITY OFFER  Good supply and ready access to sensimotor recreational items | Disabling  Enabling | Suggested  Studied, confirmed |
| Buettner and Fitzsimmons, 2003^20^ | Study on activity calendar offerings, leisure, preference and actual involvement | Case control study | 107 | RESIDENTS  Higher cognitive impairment  Higher ADL dependency  CARE CULTURE  Psychotropic drug use  ACTIVITY OFFER  Use of standard activity schedules instead of individualized activities | Disabling  Disabling  disabling  disabling | Studied, confirmed  Studied, confirmed  Studied, confirmed  Studied, confirmed |
| Brooker and Woolley, 2007; Brooker, Woolley and Lee, 2007^4^ | Study on the effect of the Enriched opportunities program (sustainable multi-level activity based model of care) | Repeated measures within subjects design | 115 | STAFF RATIO  Instability of staff  CARE CULTURE  Person-centered care  Strong management and leadership  PHYSICAL ENVIRONMENT  Communal space and equipment  Getting out of the facility  Individual assessment of abilities, life history, personality and interests  Individualized simple and fun activity and occupation  Staff training  Senior staff role ensuring residents reach potential wellbeing | disabling  Enabling  Enabling  Enabling  Enabling  Enabling  Enabling  Enabling  Enabling | suggested  Studied, *confirmed**  Studied, *confirmed**  Studied, *confirmed**  Studied, *confirmed**  Studied, *confirmed**  studied, *confirmed**  Studied, *confirmed**  Studied, *confirmed** |
| Cohen-Mansfield et al., 2009 ^24^ | Study on impact personal characteristics on engagement | cross sectional study | 193 | RESIDENTS  Female gender  Higher comorbidity Index  Higher ADL dependence  Higher cognitive functioning  Speech clarity  Making self understood  Poor hearing  CARE CULTURE  Higher number of medications  ACTIVITY OFFER  More activities of past interest to participant | Enabling  Disabling  Disabling  disabling  Enabling  Enabling  Disabling  disabling  enabling | Studied, trend  Studied, confirmed  Studied, confirmed  Studied, confirmed  Studied, confirmed  Studied, confirmed  Studied, n.s.  Studied, n.s.  Studied, confirmed |
| Cohen-Mansfield *et al.,* 2010 ^46^ | Study to determine which stimuli are engaging, refused and appropriate | Experimental study | 193 | ACTIVITY OFFER  Adjustment of type of stimuli to gender, cognitive function  Real stimuli, representative of real world tasks, social stimuli and task oriented activities | Enabling  Enabling | Studied, confirmed  Studied, confirmed |
| Cohen-Mansfield *et al.*, 2010 ^47^ | Study on impact of setting characteristics and presentation effects on engagement with stimuli | Intervention study | 193 | PHYSICAL ENVIRONMENT  Normal or bright light (not dark)  Moderate levels of sound  Presence of small group of people (4-9)  ACTIVITY OFFER  Modeling of appropriate behavior  Activities in the afternoon instead of morning | Enabling  Enabling  Enabling  Enabling  Enabling | Studied, confirmed  Studied, confirmed  Studied, confirmed  Studied, confirmed  Studied, confirmed |
| Dobbs *et al.*, 2005 ^17^ | Study on characteristics associated with lower activity involvement | cross sectional study | 421 | RESIDENTS  Depression  behavioral symptoms  pain  immobility  ADL dependency  low food intake  cognition  STAFF PERCEPTIONS  Feeling of competence to provide suited activities  PHYSICAL ENVIRONMENT  For profit organization  Smaller size  CARE CULTURE  Family involved in assessment activities  Staff assessment of activity abilities and preferences  ACTIVITY OFFER  Family involvement (hr/week)  Higher activity provision level  Staff encouragement of activity involvement  Training to facilitate activity provision | Disabling  Disabling  Disabling  Disabling  Disabling  Disabling  Disabling  Enabling  Enabling  Enabling  Enabling  Enabling  Enabling  Enabling  Enabling  Enabling | Studied, n.s.  Studied, n.s.  Studied, n.s.  Studied, n.s.  Studied, confirmed  Studied, n.s.  Studied, confirmed  Studied, n.s.  Studied, n.s.  Studied, n.s.  Studied, confirmed  Studied, n.s.  Studied, confirmed  Studied, n.s.  Studied, confirmed  Studied, n.s. |
| Ducak, Denton and Elliot, 2016 ^28^ | Study on factors that affected implementation of Montessori Methods for Dementia in Canadian long term care homes | Qualitative study using semi structured telephone interviews | 17 recreation staff and multidisciplinary consultants’ | STAFF PERCEPTIONS  Support of manager and colleagues  STAFF RATIO  Limited resources  CARE CULTURE  Task oriented work environment  Limited knowledge on beneficial effects of activities of staff  ACTIVITY OFFER  Activities provided by recreational staff  OTHER  Restrictive rules and regulations of government | Enabling  Disabling  Disabling  Disabling  Disabling  disabling | Study result  Study result  Study result  Study result  Study result  Study result |
| Edvardsson, 2013 ^37^ | Study on the relationship between percent centered care, quality of life and participation in everyday activities | cross sectional study | 1266 | RESIDENTS  Higher cognitive scores  STAFF PERCEPTIONS  Perceived high workload  CARE CULTURE  Person centered care  Task oriented working / inflexibility  Biographical and functional knowledge of residents | Enabling  Disabling  Enabling  Disabling  Enabling | Studied, confirmed  Suggested  Studied, confirmed  Suggested  Suggested |
| Fleming and Purandare, 2010 ^41^ | Study to identify which environmental recommendations should be used in the design of long term care facilities for persons with dementia | Literature review | 148 relevant articles | PHYSICAL ENVIRONMENT  Homelikeness and presence of outside space, when combined with staff interaction / encouragement | Enabling | Studied, confirmed |
| Green and Cooper, 2000 ^36^ | Study on factors that contribute most to occupational performance in nursing homes (not dementia specific) | Qualitative study using semi- structured interviews | 20 care managers | RESIDENTS  Residents ability and level of motivation  CARE CULTURE  Strong leadership  Philosophy of care  Organizational routines limiting autonomy of residents  ACTIVITY OFFER  Residents control over and choice of activity  Recognition of value of ‘normal activities’ | Enabling  Enabling  Enabling  Disabling  Enabling  Enabling | Study result  Study result  Study result  Study result  Study result  Study result |
| Hancock *et al.,* 2006 ^9^ | Study to identify unmet needs or people with dementia and characteristics associated with high levels of unmet needs.  Study outcome is score on CANE scale of unmet needs, with stimulating daytime activities as most commonly scored unmet need. | cross sectional study | 238 | RESIDENTS  Behavioral problems  Younger age  Shorter length of stay  Depression  Anxiety | Disabling  Disabling  Disabling  Disabling  Disabling | Studied, confirmed  Studied, confirmed  Studied, confirmed  Studied, confirmed  Studied, confirmed |
| Harmer and Orrell, 2008 ^27^ | Study on concept of meaningful activity as defined by people with dementia in care homes, staff and family caregivers | Qualitative study using focus group interviews | 17 residents  15 staff  8 family | RESIDENTS  Loss of abilities due to cognitive impairment  Communication problems in ethnic minority groups  Lack of motivation / loss of interest  STAFF RATIO  Lack of skills  Lack of sufficient staff  CARE CULTURE  Prioritization of physical over psychosocial needs  Lack of knowledge on importance of engagement in activities  ACTIVITY OFFER  Suited activity offer adjusted to skills and preference  Lack of organized activities  Activity provision not restricted to activity workers | Disabling  Disabling  Disabling  Disabling  Disabling  Disabling  Disabling  Enabling  disabling  Enabling | Study result  Study result  Study result  Study result  Study result  Study result  Study result  Study result  Study result  Study result |
| Kolanowski *et al.*, 2006 ^18^ | Study on predictors of time of engagement and participation in activities matched to skill level, activities that matched interests, and both | Cross over experimental | 30 | RESIDENTS  Agitation  Passivity  Higher MMSE score  ADL dependency  CARE CULTURE  (Psychotropic) medication use | Disabling  Disabling  Enabling  Disabling  Disabling | Studied, n.s.  Studied, n.s.  Studied, confirmed  Studied, confirmed  Studied, n.s. |
| Kolanowski et al., 2009 ^32^ | Study on the anticholinergic burden of nursing home residents with dementia and the association between use of anticholinergic drugs and activity engagement. | Cross sectional study | 116 | RESIDENTS  Non-treated depression  STAFF RATIO  Low staff ratio  CARE CULTURE  Anticholinergic drug use  ACTIVITY OFFER  Poor quality of activity offer | Disabling  Disabling  Disabling  disabling | Suggested  Suggested  Studied, n.s.  suggested |
| Kolanowski, 2011 ^48^ | Study on effect of theory based activity intervention on behavioral symptoms | Randomized controlled trial | 128 | ACTIVITY OFFER  Activities adjusted on skill level and personal interests | Enabling | Studied, confirmed |
| Kolanowski *et al.*, 2012 ^25^ | Study on the relationship between mood and personality factors and attention, time on task and disengagement in an activity intervention | Randomized controlled trial | 128 | RESIDENTS  Positive self-rated mood  Neuroticism  conscientiousness | Enabling  Disabling  Enabling | Studied, confirmed  Studied, n.s.  Studied, n.s. |
| Kuhn, Fulton & Edelman*,* 2004 ^15^ | Study on the influence on cognitive and functional impairment and care setting on participation in activities | Dementia care mapping observations | 166 | RESIDENTS  Cognitive impairment  Functional impairment  STAFF PERCEPTIONS  Lack of skills  CARE CULTURE  Psychotropic drug use  ACTIVITY OFFER  Large group activities | Disabling  Disabling  Disabling  Disabling  disabling | Studied, *confirmed*  Studied, *confirmed*  Suggested  Suggested  Suggested |
| LeBlanc *et al.*, 2006 ^49^ | Study on the effect of 4 versions of a 2-choice preference assessment on engagement | Case control study | 4 | ACTIVITY OFFER  Offering activity choices | Enabling | Study result |
| Morgan-Brown, Newton and Ormerod, 2013 ^45^ | Study on social engagement and interactive occupation before and after conversion of nursing homes to a household model environment | Observational study using snapshot observation method |  | PHYSICAL ENVIRONMENT  Household environment | enabling | Studied, confirmed |
| Nolan, Grant and Nolan, 1995 ^50^ | Study on interaction and activity levels amongst respite, long stay and short stay patients in two hospital settings and beliefs of care staff. | Mixed methods of naturalistic field observation and staff questionnaires | 49 residents  24 staff | ACTIVITY OFFER  Activity workers solely provide activities  Activity provision is not seen as part of role care staff | disabling  disabling | Study result  Study result |
| den Ouden et al., 2015 ^12^ | Study to provide insight into the daily activities of psychogeriatric and somatic nursing home residents and body positions during these activities | Cross-sectional observation study | 723 | STAFF PERCEPTIONS  High workload  Negative work experience  STAFF RATIO  Amount of staff  Education level  CARE CULTURE  Negative attitude toward activities  ACTIVITY OFFER  Lack of relevant and meaningful activities  PHYSICAL ENVIRONMENT  Occupation stimulating environment | Disabling  Disabling  Enabling  Enabling  Disabling  Disabling  enabling | Suggested  Suggested  Suggested  Suggested  Suggested  Suggested  Suggested |
| Orsulic-Jenas, Judge and Camp, 2000 ^86^ | Study on the effect of Montessori-based activities on engagement and affect | Experimental case-control | 16 | ACTIVITY OFFER  Large group activity offer  Individual and small group activity offer | Disabling  Enabling | Suggested  Study result |
| Perrin, 1997 ^34^ | Study to identify occupational need in severe dementia | Dementia care mapping observations | 109 | STAFF RATIO  Lack of skills  Insufficient knowledge of dementia  CARE CULTURE  Focus on task related work | Disabling  Disabling  disabling | Suggestion  Suggestion  Suggestion |
| Pulsford, 1997 ^33^ | Study on which therapeutic activities are provided, their effectiveness, and reasons why they are sometimes not offered | Literature review | No information available | STAFF PERCEPTIONS  Emotional and task related demands  lack of time  lack of skills  STAFF RATIO  Shortness of staff  CARE CULTURE  ‘not my job’  No perceived benefit from activities  Person centered care (looking at the benefits for the individual, no ‘all or nothing thinking’) | Disabling  Disabling  Disabling  disabling  Disabling  Disabling  Enabling | Study result  Study result  Study result  Study result  Study result  Study result  Study result |
| Schwarz, Chaudhury and Tofle, 2004 ^42^ | Study on the effects of design interventions in long term care facility on resident and staff perceptions of care delivery | Pretest protest behavioral mapping around altering environment, focus groups | 3 mapping periods on 20-30 residents | STAFF RATIO  High staff turnover  PHYSICAL ENVIRONMENT  Homelike environment  Gap between environment and corresponding activities  ACTIVITY OFFER  Staff straining in provision of small group activities | Disabling  Enabling  Disabling  Enabling | Suggested  Study result  Study result  Study result |
| Smit *et al.*, 2012 ^44^ | Study on the relationship between small scale care and activity involvement | Cross sectional study | 1327 | PHYSICAL ENVIRONMENT  Small scale group home living care characteristics  Smaller number of residents in facility | Enabling  disabling | Studied, confirmed  Studied, n.s. |
| Smit *et al.*, 2013 ^51^ | Study on offered types of occupation and organizational and environmental contributing study | Explorative observational study using Dementia care mapping | 57 | PHYSICAL ENVIRONMENT  Homelike atmosphere  Social interaction enhancing environment  ACTIVITY OFFER  Absence of central activity program | Enabling  Enabling  Enabling | Study result  Study result  Study result |
| Tak et al., 2015 ^23^ | Study on types of activity involvement and barriers to activities as perceived by nursing home residents with dementia. | Qualitative study with short open ended interviews and in depth interviews | 37 | RESIDENTS  Physical limitations (e.g. back problems, hearing, eye sight)  PHYSICAL ENVIRONMENT  Accurate light and sound levels  ACTIVITY OFFER  Availability of various, sufficient and ongoing activities  Resources for activities (space, transportation, materials)  Meaningful activities tailored to needs, preferences and capacities  Involving residents in planning of activities  Up to date activity plans for each resident | Disabling  Enabling  Enabling  Enabling  Enabling  Enabling  enabling | Study result  Study result  Study result  Study result  Study result  Study result  Study result |
| Train *et al.*, 2005 ^35^ | Study to explore positive and negative experiences of family carers, staff and people with dementia in long term care settings | Qualitative study with semi structured interviews | 21 residents, 17 relatives, 30 staff | STAFF PERCEPTIONS  Work load – lack of time  STAFF RATIO  Leave of skilled staff that were not replaced  ACTIVITY OFFER  Tailored activities to individual needs instead of group activities | disabling  Disabling  Enabling | Study result  Study result  Study result |
| Verbeek *et al.*, 2010 ^43^ | Study on the effects of small scale living dementia care facilities on residents, family caregivers and staff | Quasi experimental study | 259 residents | PHYSICAL ENVIRONMENT  Small scale living facility | Enabling | Studied, significant |
| Voelkl, Fries, and Galecki, 1995 ^22^ | Study on predictors of nursing home residents (with and without cognitive problems) participation in activity programs based on Lawtons model on antecedents and consequences of older adults’ activity participation | Cross sectional study | 2.672 | RESIDENTS  Use of care resources  Female gender  Better cognition  Depression  Sense of initiative  ACTIVITY OFFER  Activity repertoire / preference  Location preferences – preference to stay in room | Disabling  Enabling  Mixed  Disabling  Enabling  Enabling  Disabling | Studied, significant  Studied, significant  Studied, significant  Studied, significant  Studied, significant  Studied, significant  Studied, significant |
| Voelkl, Winkelhake, Jeffries and Yoshioka, 2003 ^21^ | Study on the use of the nursing home environment of residents and staff, and on staff perceptions on predictors, barriers and affordances of residents’ engagement in activities. | Case study using observations and focus group interviews with staff | 42 residents  12 staff | RESIDENTS  Better cognition  Better physical abilities  Personal preferences  Resident perceptions such as shame  Lack of communication abilities  Depression  STAFF RATIO  Staff turnover  CARE CULTURE  Task oriented staff philosophy  ACTIVITY OFFER  Variety in activities  Lack of recreational resources  Routine  Difficult working relationship care and recreational staff  PHYSICAL ENVIRONMENT  Physical environment – inviting environment for residents, appropriate furniture. Adjusted to needs and preferences | Enabling  Enabling  Enabling  Disabling  Disabling  Disabling  Disabling  Disabling  Enabling  Disabling  Disabling  Disabling  Enabling | Study result  Study result  Study result  Study result  Study result  Study result  Study result  Study result  Study result  Study result  Study result  Study result  Study result |
| Vollicer *et al.*, 2006 ^3^ | Study on the effects of continuous activity programming on behavioral problems | Observational | 90 | STAFF RATIO  Low staff ratio  ACTIVITY OFFER  Knowledge on activity provision  Activity provision by all staff, not only activity staff  Continuous activity program instead of traditional activity schedules | Disabling  Enabling  Enabling  Enabling | Studied, confirmed  Study result  Study result  Studied, confirmed |
| Wood *et al.,* 2005 ^87^ | Study on routine activity situations on an Alzheimer’s disease special care unit concerning time use, interactions and affect | Case study | 7 | CARE CULTURE  Skills of staff to recognize activity need  PHYSICAL ENVIRONMENT  Background media  Making use of activating environment | Enabling  Disabling  Enabling | Study result  Study result  Study result |
| Wood, Womack and Hooper, 2009 ^38^ | Study to explore interrelationships among routine activity situations on 2 SCUs and daily time use and emotional wellbeing | Case study | 14 | CARE CULTURE  Lack of understanding importance of occupation  Insufficient attention to resident’s occupational needs, initiatives and capabilities  Low levels of staff-resident interactions  PHYSICAL ENVIRONMENT  Background media  ACTIVITY OFFER  Focus on activity schedules instead of everyday occupation  Absence of activity staff | Disabling  disabling  disabling  Disabling  Disabling  Disabling | Study result  Study result  Study result  Study result  Study result  Study result |
| Zimmerman *et al.,* 2005 ^16^ | Study on which elements of long term dementia care are related to resident quality of life. Outcomes reported: higher score on QoL in AD-Activity and / or Type 1 behavior category codes considering to be good behaviors in DCM | Cross sectional study in 45 facilities using patient reports, staff reports and dementia care mapping observations | 421 | RESIDENTS  Age  Gender  Race  Marital status  Length of stay < 12 months  Better cognitive status  No behavioral symptoms  Less functional impairment  Comorbidity  Depression  Ungroomed or inappropriate appearance  STAFF PERCEPTIONS  Work satisfaction  Work stress  Perceived success in treatment of residents  STAFF RATIO  Stability of provider-resident assignments  Staff turnover  Ratio receiving professional or unprofessional treatment  Formal staff training  CARE CULTURE  Approach to people with dementia  Policies and practices (policies on admission, discharge, and problematic behavior)  Involvement of physicians or paramedics in care planning  Resident assessment  Positive communication / interaction with staff / physical contact / positive person work  Use of cholinesterase inhibitor  Use of antipsychotic or sedative hypnotic  Observed in restraints  ENVIRONMENT  Facility type (traditional or new model, large or small scale, for or non-profit)  ACTIVITY OFFER  Family involvement (hr / week)  Specialized worker perspective  Encourage activities  Use of stimuli | Neutral  Neutral  Neutral  Neutral  Enabling  Enabling  Enabling  Enabling  disabling  disabling  disabling  enabling  disabling  Enabling  disabling  disabling  enabling  Enabling  enabling  Disabling  Enabling  Enabling  Enabling  enabling  enabling  disabling  Neutral  Enabling  Disabling  Enabling  Enabling | Studied, n.s.  Studied, n.s.  Studied, n.s.  Studied, n.s.  Studied, confirmed  Studied, confirmed  Studied, confirmed  Studied, confirmed  Studied, n.s.  Studied, confirmed  Studied, confirmed  Studied, confirmed  Studied, n.s.  Studied, confirmed  Studied, confirmed  Studied, confirmed  Studied, confirmed  Studied, n.s.  Studied, confirmed  Studied, n.s.  Studied, n.s.  Studied, confirmed  Studied, disproved  Studied, confirmed  Studied, confirmed  Studied, confirmed  Studied, confirmed  Studied, n.s.  Studied, n.s.  Studied, n.s.  Studied, n.s. |

* these factors are components of the Enriching opportunities program. The program as a whole had positive effects on activity involvement. However, the different components of the intervention were not studied separately so it is uncertain whether or not all these components indeed contribute to higher activity involvement.
