# Supplementary material for: Predictors of activity involvement in dementia care homes: a cross-sectional study
Source: BMC Geriatr. 2017 Aug 4;17:175. doi: 10.1186/s12877-017-0564-7 (PMC5545000; doi:10.1186/s12877-017-0564-7)
Supplement: Supplementary file 2 — Family caregiver questionnaire. Measures and operationalization of standardized questionnaire for family caregivers in the second measurement cycle of the LAD-study [88, 89]. (DOCX 12 kb) [file 12877_2017_564_MOESM2_ESM.docx]

**Family caregiver questionnaire:** Measures and operationalization of standardized questionnaire for family caregivers in the second measurement cycle of the LAD-study

| **Measure** | **Operationalization** |
| --- | --- |
| **Family caregiver characteristics** |  |
| Age | Years |
| Gender | Male or female |
| Gender of relative with dementia | Male or female |
| Educational level | Type of education and level |
| **Family caregiver satisfaction with care** |  |
| Family involvement in care (1) | Family Perception of Caregiver Role instrument^65^ |
| Family involvement in care (2) | Family perceived involvement (F-INVOLVE) and family assessment of importance (F-IMPORTANT) of their involvement in long term care^88^ |
| Stress experienced resulting from the care giving situation | Self-Perceived Pressure from Informal Care questionnaire (SPPIC)^89^ |
